# Evaluation of Psychiatric High and Intensive Care (EPHIC-study): monitoring innovative care from a value-based approach

**DOI:** 10.1192/j.eurpsy.2024.231

**Published:** 2024-08-27

**Authors:** H. Demunter, H. Jossa, K. Vandenhout, S. Claes, R. Bruffaerts

**Affiliations:** ^1^UPC KU Leuven, Kortenberg, Belgium

## Abstract

**Introduction:**

Systematic monitoring and evaluation of innovative healthcare programs are essential to develop sustainable solutions to health needs in the population (Porter & Teisberg, 2006). Development of Psychiatric High and Intensive Care Units (HIC’s) in Belgium, following the Dutch Model (van Mierlo et al., 2013), is an innovative model for patients with acute and severe psychiatric illness, resulting in potential danger. HIC aims to provide intensive, need-adapted care with interventions that reduce (perceived) coercion, focusing on participative processes and continuity of care.

**Objectives:**

(1) What are the clinical characteristics of admitted patients? (2) How does clinical symptomatology evolve during admission? (3) How do patients, relatives and caregivers experience the process of care and recovery? (4) What is the role of HIC’s in the reformed mental health care?

**Methods:**

This is an explorative, hypothesis-generating study, using a mixed-method approach, consisting of qualitative and quantitative methods against a value-based framework. Data collection lasted 18 months in the first 9 HIC’s in Belgium. Results are based on validated questionnaires completed by adult patients and their HIC caregivers at admission and discharge (N=472).

**Results:**

We provide the first, preliminary results. Suicidality, psychotic and substance-related symptoms are the most important primary symptoms. Almost 70% have 2 or more symptoms, with psychiatric comorbidity of 50%. Substance-related- and psychotic disorders are the two most common diagnoses, followed by personality disorder cluster B and depressive disorder. 83% have been in residential care in the past, of whom 87% twice or more. The median age is 36 years, but the median age of onset of mental disorders is 21 years, which equals to 15 years in mental disorder progress and comorbidity development. Over 50% meet the criteria for Severe Mental Illness and 56% are involuntary admitted. There is a high degree of unmet needs: no outpatient care is provided for one out of five prior to admission and there is a low follow-up by mobile teams prior to and after admission (around 12% each). We found significant improvements after an average stay of 22 days for aggression, suicidality and crisis (respectively decrease of 68%, 25% and 9%); readiness to change and motivation for treatment (respectively increase of 5% and 14%) The Client Satisfaction Questionnaire scores range from 1 to 4, with an average score 3.15 out of 4.

**Conclusions:**

Based on these preliminary results we can conclude that aggression, suicidality, crisis, readiness to change and motivation for treatment all improve significantly after a short stay of 3 weeks. Despite a vulnerable, severely distressed population, patients are generally satisfied with received care. There is a high degree of unmet needs: insufficient provided outpatient care and low follow up by mobile teams.

**Disclosure of Interest:**

None Declared

